# Physical Growth Status and Feeding Methods of Chinese Infants With Cleft Lip With or Without Cleft Palate Under 1 Year of Age

**DOI:** 10.3389/fped.2020.00194

**Published:** 2020-04-21

**Authors:** Wenli Wu, Jing Sun, Huiyan Liu, Biting Chen, Zijun Gao, Yiyang Chen, Fan Li, Hongtao Wang

**Affiliations:** ^1^Department of Oral and Maxillofacial Surgery, Stomatology Medical Center, Guangzhou Women and Children's Medical Center, Guangzhou, China; ^2^Department of Clinical Nutrition, Guangzhou Women and Children's Medical Center, Guangzhou, China; ^3^Department of Child Health Care, Guangzhou Women and Children's Medical Center, Guangzhou, China

**Keywords:** cleft lip with or without palate, weight-for-age, length-for-age, body mass index-for-age (BMI), physical growth, infant

## Abstract

**Objectives:** Malnutrition is a characteristic feature of cleft lip with or without palate (CL/P). This study aims to retrospectively quantify the physical growth status, evaluate the feeding methods, and identify the possible correlation of CL/P types with growth failure.

**Methods:** The length and weight of 508 infants with CL/P and 118 healthy infants were recorded at the date of admission. The weight-for-age (W/A), length-for-age (L/A), and body mass index-for-age (BMI) of the infants were calculated.

**Results:** The L/A values were significantly lower in the infants with cleft lip (CL, 123 cases) and cleft lip with palate (CLP, 122 cases) than those of the control infants (*p* < 0.01). The W/A values of the infants with CP (263 cases) and CLP were significantly lower than those of the control infants (*p* < 0.05). However, the BMI index was not significantly different between any of the studied groups and the controls. In the mixed feeding group, the infants with CL and CP showed significantly lower L/A (*p* < 0.05) and W/A (*p* < 0.05), respectively.

**Conclusion:** Physical growth issues were more common in the infants with CP and CLP. Because this was a retrospective study, the foods supplied to the patients were not strictly uniform, therefore, a prospective study with unified food supplement may be needed to confirm these findings.

## Introduction

Cleft lip with or without palate (CL/P) patients are a group of congenital maxillofacial malformations that include patients with cleft lip only (CL), cleft palate only (CP), and cleft lip together with cleft palate (CLP) (1). In developed countries, the incidence of CL/P is around 1–2/1,000 of births (2), while the incidence rate of CL and CLP in China are 5.03/10,000 and 8.97/10,000, respectively (3). The prevalence of CL/P in Shanghai in China is 9.37/10,000 (4). Approximately 3,800 patients with CL/P died globally in 1980–2017 (5). Due to the presence of palate and/or maxillary defects before cleft-restoration surgery, these patients often have feeding issues and airway infections, resulting in malnutrition and developmental delays, including slow growth and weight gain.

Malnutrition is a characteristic feature of CL/P (6, 7). The growth pattern of the majority of patients with CL/P appears to be markedly abnormal, while some patients still follow a relatively normal pattern of weight and length acquisition (8–11). In the past decade, the world has undergone vast changes. Economic progression has led to changes in people's lifestyle, health awareness, as well as great improvement in nutritional supplement composition. Nevertheless, the majority of our understanding of malnutrition and developmental delay in infants with CL/P has been acquired over the past 10 years (8–11). Very little novel statistical data regarding the true development status of infants with CL/P has been published, with the exception of the studies from and Prahl et al. (12) and Miranda et al. (7). There is an urgent need for a large case statistical investigation of malnutrition and developmental delay in infants with CL/P in other districts and nations, especially in China.

Physical growth reflects the health outcome of infants. Insufficient physical growth may reflect abnormal conditions, such as inadequate nutrition and chronic health conditions, resulting in development disorders of systemic organs. This study aims to retrospectively quantify the growth characteristics and nutritional status of 508 Chinese infants with CL/P before the performance of primary CL/CP repairs and to identify the potential correlations between feeding methods or CL/P types and patient growth failure. These results will help to achieve the best condition for infants with CL/P, to enable a faster recovery and a reduced incidence of complications before repair surgeries.

## Methods

### Patients

Five hundred and eight children with CL/P were enrolled in this retrospective study at the Department of Oral and Maxillofacial Surgery, Guangzhou Women and Children's Medical Center in Guangzhou, China between January and December 2017. The participants were 3–12 months of age at the time of admission. Participants were divided into CL [123, male/female (M/F): 74/49], CP (263, M/F: 106/157), and CLP (122, M/F: 87/35) groups. Infants with associated chronic diseases, genetic syndromes, or other malformations were not included in this study. There were 16 cases of premature infants (3.15%). In 32 cases (6.3%) the mother had a disease during pregnancy, including diabetes (71.88%, 23/32), hypertension (12.5%, 4/32), and diabetes with hypertension (6.25%, 2/32). This study was approved by the Institutional Review Board of Guangzhou Women and Children's Medical Center. All of the procedures performed in studies involving human participants were in accordance with the ethical standards of the institutional and national research committee and with the 1964 Helsinki declaration and its later amendments or comparable ethical standards. Written informed consent was obtained from the guardians of all individual participants included in the study.

### Clinical Information Records

The clinical information of each patient was recorded in a nursing record system (actually, an electronic medical record system) before surgery, including regular physical examination, feeding method and history, previous surgical records, diseases history records.

### Growth Measurements

The indices of age, weight, and body length before repair surgery, feeding methods (including breast-feeding alone, formula-feeding alone, and mixed-feeding, which included a combination of breast- and formula-feeding), and the timings of food supplements administrations were collected. Indices of length-for-age (L/A), weight-for-age (W/A), and body mass index (BMI) were used to evaluate the physical growth status of the infants (13). Standard Infant and Toddler scales (Seca 376, Germany) were used to measure the weight of the babies. The body length was detected using a horizontal anthropometer (Seca 416, Germany) in the supine position. Routine physical examinations were performed on 74–118 normal infants at the Department of Nutrition for Health and Development, and the data from these participants served as the controls (Table 1).

**Table 1:** Nutritional status indices.

**Variable**	**Patient**	**Control**	***P*-value**
**CL (*****n*****)**	**123**	**77**	
Age(M)	5.16 ± 2.11	5.53 ± 2.34	0.249
Weight (kg)	7.37 ± 1.10	7.42 ± 1.28	0.811
Length (cm)	65.40 ± 4.08	65.99 ± 4.75	0.354
BMI (kg/m^2^)	17.28 ± 1.95	16.92 ± 1.30	0.163
Z-score			
W/A	−0.09 ± 1.03	0.12 ± 0.91	0.143
L/A	−0.24 ± 1.33	0.24 ± 0.95	0.006
BMI	0.07 ± 1.28	−0.03 ± 0.89	0.516
**CP (*****n*****)**	**263**	**74**	
Age(M)	8.36 ± 1.59	8.43 ± 2.26	0.746
Weight (kg)	8.20 ± 0.96	8.47 ± 1.12	0.039
Length (cm)	70.22 ± 3.74	70.69 ± 3.86	0.345
BMI (kg/m^2^)	16.77 ± 1.93	16.90 ± 1.20	0.600
Z-score			
W/A	−0.28 ± 1.07	0.04 ± 0.85	0.019
L/A	−0.17 ± 1.35	0.12 ± 0.97	0.080
BMI	−0.24 ± 1.50	−0.05 ± 0.83	0.298
**CLP (*****n*****)**	**122**	**118**	
Age(M)	7.08 ± 2.35	7.47 ± 3.37	0.295
Weight (kg)	7.81 ± 1.06	8.06 ± 1.46	0.137
Length (cm)	68.23 ± 4.92	68.87 ± 5.79	0.353
BMI (kg/m^2^)	16.79 ± 1.67	16.87 ± 1.24	0.682
Z-score			
W/A	−0.44 ± 0.89	0.07 ± 0.86	0.000
L/A	−0.42 ± 1.50	0.13 ± 0.98	0.001
BMI	−0.26 ± 1.17	−0.01 ± 0.84	0.058

*Age, surgery age (month); M, month; W/A, Weight- for-Age; L/A, Length -for-Age; BMI, Body Mass Index-for-Age; p < 0.05 are considered statistically significant*.

### Statistical Analyses

L/A and W/A *z*-scores were calculated using the WHO Child Growth Standards (13). L/A (<12 months) and W/A (<12 months) were estimated by comparing to the growth charts of WHO Child Growth Standards (13). The standardized growth data of WHO Child Growth Standards (13) was used for the BMI *z*-scores calculation. Descriptive statistics, such as means, standard deviations (SDs) were used to summarize the data. All statistical analyses were conducted using SPSS version 22.0 (IBM SPSS Statistics, US). *P*-values were two-sided and *p*-values of <0.05 were considered to be statistically significant.

## Results

### Indices of Nutritional Status in Infants With CL, CP, and CLP

The mean age, weight, length, and BMI of the patients with CL were not significantly different to those of controls. The *z*-scores and *p*-value of the mean L/A in infant with CL were significantly lower than those of the control infants (Table 1).

The mean weight of the 263 infants with CP was significantly lower than that of the controls (*p* < 0.05). The *z*-scores and *p*-value of the mean W/A in the infants with CP were significantly lower than those of the control group (*p* < 0.05). The W/A in the infants with CP was lower than that of the control group (*p* < 0.05). Other indexes, including age, length, BMI, and L/A, were no difference between the CP and control groups (Table 1).

The infants with CLP group contained 122 patients, including those chosen for repair in one stage (closure of the lip, hard palate and soft palate simultaneously) and in two stages (closure of lip first, and closure of hard palate and soft palate few months later). The *z*-score and *p*-value of both mean W/A and L/A in the infants with CLP were significantly lower than those of the healthy controls (*p* < 0.001) (Table 1). The mean age of the infants (42/122) who were chosen for repair in two stages was significantly lower than those who was chosen for repair in one stage at the initial surgery (*p* < 0.01). However, no difference was found in any of the nutritional indices between the groups.

### Effect of Feeding Method on the Nutritional Status of Infants With CL, CP, and CLP

The age of the breast-feeding group in the CL infants was significantly lower than that of the mixed feeding (*p* < 0.05) and formula-feeding groups (*p* < 0.05), respectively. In terms of body length, the *z*-score of the L/A in the mixed-feeding group was lower than that of the breast-feeding (*p* < 0.05) and formula-feeding (*p* < 0.05) groups, separately (Table 2).

**Table 2:** Nutritional indices in the different feeding groups.

**Variable**	**Breast-feeding^**a**^**	**Mixed-feeding^**b**^**	**Formula-fed^**c**^**	***P*-value**	**P^**a–b**^**	**P^**a–c**^**	**P^**b–c**^**
**CL** ***(n)***	**33**	**42**	**47**				
Age (M)	4.33 ± 1.41	5.50 ± 2.13	5.49 ± 2.37	0.025	0.017	0.015	0.981
Z-score							
W/A	0.05 ± 1.30	−0.37 ± 0.88	0.05 ± 0.88	0.101			
L/A	0.08 ± 1.43	−0.68 ± 1.26	−0.09 ± 1.25	0.029	0.014	0.557	0.038
BMI	0.01 ± 1.39	0.03 ± 1.38	0.14 ± 1.11	0.885			
**CP (*****n*****)**	**19**	**154**	**90**				
Age(M)	7.53 ± 1.54	8.55 ± 1.60	8.26 ± 1.56	0.022	0.009	0.069	0.168
Z-score							
W/A	0.21 ± 0.84	−0.33 ± 1.12	−0.32 ± 1.02	0.116			
L/A	0.01 ± 1.26	−0.15 ± 1.29	−0.22 ± 1.44	0.793			
BMI	0.27 ± 1.36	−0.31 ± 1.62	−0.26 ± 1.30	0.282			
**CLP (*****n*****)**	**11**	**75**	**36**				
Age(M)	5.18 ± 2.60	7.31 ± 2.27	7.19 ± 2.24	0.017	0.005	0.012	0.809
Z-score							
W/A	−0.27 ± 1.04	−0.45 ± 0.88	−0.48 ± 0.89	0.779			
L/A	−0.05 ± 1.51	−0.33 ± 1.58	−0.72 ± 1.31	0.300			
BMI	−0.35 ± 1.21	−0.34 ± 1.19	−0.08 ± 1.12	0.542			

Age, surgery age (month); M, month; W/A, Weight-for-Age; L/A, Length -for-Age; BMI, Body Mass Index-for-Age; p < 0.05 are considered statistically significant.

a means group of breast-feeding;

b means group of mixed-feeding;

c means group of formula-fed;

a−b means comparison between groups of breast-feeding and mixed-feeding;

a−c means comparison between groups of breast-feeding and formula-fed;

b−c *means comparison between groups of mixed-feeding and formula-fed*.

The age of the breast-feeding group in the CP infants was significantly lower than those of the mixed-feeding group (*p* < 0.01) and formula-feeding groups (*p* < 0.05), respectively. In terms of body weight, the *z*-score of the W/A in the breast-feeding group was significantly higher than those of the other two groups (*p* < 0.05; Table 2).

The mean age of the breast-feeding group in the CLP infants was lower than that of the mixed- and formula-feeding groups (*p* < 0.01 and *p* < 0.05, respectively; Table 2).

### Effect of Semi-solid Food on the Nutritional Status of Infants With CP and CLP

The mean age of the CL group (5.16 months) was below the WHO-recommended age for the addition of complementary food to the diet (6 months) (14). The influence of supplemented complementary food on the nutritional status of CL preoperatively will not be discussed here. The mean age of the CP patients who added complementary food to the diet was over 6 months. To investigate whether the addition of semi-solid food effects the growth of infants with CP, we compared the weight and length of the infants with CP who were supplied with semi-solid food to those who were not supplied with semi-solid food (Table 3). The mean age of the infants supplied with semi-solid food supplements was higher those without semi-solid food (8.65 ± 1.49 vs. 7.76 ± 1.66, *p* < 0.01). The *z*-scores and *P*-value of the mean W/A were significantly lower in the infants with CP supplied with semi-solid food than those without semi-solid food (*p* < 0.01), respectively. These results suggested that the supplementation of semi-solid food to the diet prior to cleft palate repair might influence weight gain. Because this was a retrospective study, the reasons behind the parent's decision to choose semi-solid food or not is not clear and, thus, this result needs to be further verified in a prospective study (Table 3).

**Table 3:** Nutritional indices in added and not added semi-solid food supplementation groups.

**Variable**	**Added**	**Not added**	***P*-value**
**CP (*****n*****)**	**179**	**84**	
Age(M)	8.65 ± 1.49	7.76 ± 1.66	0.000
*Z*-score			
W/A	−0.43 ± 1.12	0.03 ± 0.89	0.001
L/A	−0.27 ± 1.35	0.03 ± 1.33	0.095
BMI	−0.36 ± 1.64	0.02 ± 1.14	0.053
**CLP (*****n*****)**	**53**	**49**	
Age(M)	8.11 ± 1.74	7.51 ± 1.86	0.094
Weight (kg)	8.14 ± 0.99	8.05 ± 0.76	0.594
Length (cm)	70.08 ± 4.97	68.79 ± 3.31	0.129
*Z*-score			
W/A	−0.45 ± 0.85	−0.39 ± 0.95	0.751
L/A	−0.28 ± 1.70	−0.53 ± 1.28	0.396
BMI	−0.37 ± 1.13	−0.11 ± 1.23	0.268

*Age, surgery age (month); M, month; W/A, Weight-for-Age; L/A, Length -for-Age; BMI, Body Mass Index-for-Age; p < 0.05 are considered statistically significant*.

## Discussion

W/A and L/A were introduced as novel physical growth indices in 2006 (13). These indices reveal the physical growth status more precisely than the previous indices because they are calculated based on age. Historically, much of the results regarding the physical growth impact of CL/P has been calculated using the data from a decade ago, all of which were calculated using the old indices of average weight and average length (7–10). In this retrospective study, we quantified the physical growth status of 508 Chinese infants under 1 year of age that had CL/P and analyzed the potential correlations between the feeding methods in the different CL/P sub types. The results show that the L/A were significantly lower in infants with CL than those observed in controls of similar ages (*p* < 0.01). The W/A of the infants with CP were significantly lower than that of the controls (*p* < 0.05). However, the L/A in the CL group and the W/A in the CP group were still within the normal range, according to the Baby Growth Chart. The L/A and W/A were both significantly lower in the infants with CLP than in the controls (*p* < 0.01).

Among the three feeding methods, the CL infants in the mixed-feeding group had the lowest L/A level (*p* < 0.05). The CL infants in the breast-feeding and formula-feeding group did not show obvious differences in physical traits preoperatively. The *z* score of W/A was the highest in the CP infants of breast-feeding group (*p* < 0.05). However, there were no statistical difference in the physical stature among the infants with CLP in the three groups. Conversely, in the infants with CL/P, the infants with breast-fed showed an improvement in the measurements of physical development, while, the mixed-fed patients showed a relative delay in the development of the physical indices. However, there was little difference between the measurements of the three groups, thus, for the infants with CL/P, especially those with CLP, who are unable to reach the pharyngeal closed state and have insufficient sucking power, the feeding method may not be a significant factor in the physical growth.

Using old indices (average weight and average length), the findings of previous studies investigating physical growth in infants with CL/P were quite consistent. These studies found that most physical growth indices were lower in infants with CL/P than those observed in normal controls. A study from Bowers group showed that the difference in physical growth indices were less significant in infants with CL compared to infants with CP and CLP. However, the body length was significantly lower in the infants with CP and CLP than those of the controls (8). Conversely, the data from Felix-Schollaart (9) and Cunningham (10) indicate that the height of all infants with CL/P is lower than that of control infants. Miranda et al. (7) used W/A and L/A as the indices to investigate the impact of CL/P on infant physical growth in 2016 in Brazil. Miranda and colleagues suggested that infants with both CL and CP had lower W/A and BMI than the normal controls. Many factors may influence the physical growth of infants with CL/P, including recurrent respiratory infections and feeding problems. Previous studies have indicated that issues in physical growth are more frequent and severe in the early infancy of patients with CL/P, CP, or CLP (9, 15, 16). The reason for this difference might be that there is an anatomical defect between the oral and nasal cavities in both infants with CP and those with CLP, which could impair the ability to create a suction vacuum, resulting in the leakage of milk as well as frequently causing airway infections in infants with CLP below the age of 3 months (9). These findings are consistent with our results, which show that the L/A and W/A are lower in infants with CLP than those in control infants, while only the L/A in the infants with CL and the W/A in the infants with CP were lower than those observed in the control group.

In terms of the feeding method, Gopinath et al. indicated that a higher percentage of breast-fed infants with CLP had normal physical growth than those in the normal control group (11). Our results also support this conclusion. In our analysis, we compared three different types of feeding: breast-feeding, mixed-feeding, and formula-feeding. Of the breast-fed groups, the CP infants had the highest W/A, and the CL infants had the highest L/A. Conversely, in the mix-fed groups, the lowest W/A and L/A were observed in the CL and CP infants. Breast-feeding appears to be advantageous for the infants with CL, because in these cases, the repair surgery is usually performed at an early age, at which point food supplementation may not be an issue. Our data indicate that, in patients with CL/P, especially the infants with CLP, it appears that the feeding method is not a significant factor. No differences were observed between the different feeding methods in infants with CLP. Therefore, the feeding method used may not be an important factor in infants with CLP. Indeed, although parents may insist on exclusive breast-feeding before the repair operation, there is no statistical difference between the groups underwent different feeding methods in infants with CLP.

In the analysis of the influence of semi-solid food supplementation, we only compared the CP and CLP groups, because the infants with CL were too young to supply be supplied with semi-solid food. Semi-solid food supplements are usually included in the diet of infants over 6-months of age (14). In the CP group, the mean W/A index of the infants supplemented with semi-solid food was significantly lower than those that were not supplemented with semi-solid food. This data suggests that adding semi-solid food prior to cleft palate repair may have a negative effect on physical growth in infants with CP. This effect may due to the change in eating habit, which is more tough for infants with CL/P than normal infants. However, further prospective study is necessary to verify this conclusion. There were no differences in the indices between the CLP infants that were supplemented with semi-solid food and the normal controls. Considering that all patients with CL/P are required to take liquid foods for 2–4 weeks after the repair surgery, we suggest that semi-solid food supplement is not recommend for the infants with CP or CLP, even in cases where the patient is over the age of 6 months.

Because this was a retrospective study, there are some limitations. It would be of value to validate these findings through the use of a prospective study. In a prospective study, a standardized feeding method could be implemented to reduce the possible influence of varied feeding methods. Furthermore, the levels of growth hormones in the enrolled infants should be examined to exclude the possible influence of congenital growth hormone deficiency.

Our research shows that the physical growth indices of infants with CL/P below 1 year of age are significantly different to healthy controls at a similar age, especially in infants with CP and CLP. The finding that breast-feeding is still the optimal feeding method for infants with CL/P needs to be verified by a prospective study with unified food supplement. The limitation of this study is that did not calculate the intake of the diet. The results of this study may help to provide guidance for clinicians and parents in selecting and adjusting the feeding method in cases of different sub-types CL/P.

## Data Availability Statement

The datasets generated and analyzed during the present study are available from the corresponding author on reasonable request.

## Ethics Statement

The studies involving human participants were reviewed and approved by the Institutional Review Board of Guangzhou Women and Children's Medical Center. Written informed consent to participate in this study was provided by the participants' legal guardian/next of kin.

## Author Contributions

WW and HW conceived and designed research. JS analyzed and interpreted data. HL, BC, and ZG collected data. YC and FL conducted research. WW wrote the initial paper. HW revised the paper. WW had primary responsibility for final content. All authors read and approved the final manuscript.

## Conflict of Interest

The authors declare that the research was conducted in the absence of any commercial or financial relationships that could be construed as a potential conflict of interest.

## Abbreviations

BMI: body mass index-for-age
CI: confidence intervals
CL: cleft lip
CLP: cleft lip with palate
CL/P: cleft lip with or without palate
L/A: length-for-age
SD: standard deviations
W/A: weight-for-age.

## References

[1] Worley ML Patel KG Kilpatrick LA. Cleft lip and palate. Clin Perinatol. (2018) 45:661–78. 10.1016/j.clp.2018.07.006 30396411

[2] Watkins SE Meyer RE Strauss RP Aylsworth AS. Classification, epidemiology, and genetics of orofacial clefts. Clin Plast Surg. (2014) 41:149–63. 10.1016/j.cps.2013.12.003 24607185

[3] Dai L Zhu J Zhou GX Wang YP Miao L. The monitoring of cleft lip with or without cleft palate in China: 1996-2000. Zhonghua Kou Qiang Yi Xue Za Zhi. (2003) 38:438–40.3. 10.3760/j.issn:1002-0098.2003.06.013 14703478

[4] Li L Yu HT Wang XD Zhou F Wang F Wang CF. Analysis of birth defect rate trend of cleft lip and palate in Shanghai from 2007 to 2016. Zhonghua Kou Qiang Yi Xue Za Zhi. (2018) 53:301–6. 10.3760/cma.j.issn.1002-0098.2018.05.003 29972986

[5] GBD 2017 Causes of Death Collaborators Global, regional, and national age-sex-specific mortality for 282 causes of death in 195 countries and territories, 1980-2017: a systematic analysis for the Global Burden of Disease Study 2017. Lancet. (2018) 392:1736–88. 10.1016/S0140-6736(18)32203-7 30496103 PMC6227606

[6] Giridhar VU. Role of nutrition in oral and maxillofacial surgery patients. Natl J Maxillofac Surg. (2016) 7:3–9. 10.4103/0975-5950.196146 28163471 PMC5242071

[7] Miranda GS Marques IL de Barros SP Arena EP de Souza L. Weight, length, and body mass index growth of children under 2 years of age with cleft lip and palate. Cleft Palate Craniofac J. (2016) 53:264–71. 10.1597/14-003 25554856

[8] Bowers EJ Mayro RF Whitaker LA Pasquariello PS Larossa D Randall P. General body growth in children with clefts of the lip, palate, and craniofacial structure. Scand J Plast Reconstr Surg Hand Surg. (1987) 21:7–14. 10.3109/02844318709083572 3589583

[9] Felix-Schollaart B Hoeksma JB Prahl-Andersen B Growth comparison between children with cleft lip and/or palate and controls. Cleft Palate Craniofac J. (1992) 29:475–80. 10.1597/1545-1569(1992)029<0475:GCBCWC>2.3.CO;2 1472529

[10] Cunningham ML Jerome JT. Linear growth characteristics of children with cleft lip and palate. J Pediatr. (1997) 131:707–11. 10.1016/S0022-3476(97)70097-0 9403650

[11] Gopinath V Muda W. Assessment of growth and feeding practices in children with cleft lip and palate. Southeast Asian J Trop Med Public Health. (2005) 36:254–8. 15906679

[12] Prahl-Andersen B Care for children born with cleft lip, jaw and/or palate. Ned Tijdschr Tandheelkd. (2005) 112: 242–6. 16047961

[13] WHO Multicentre Growth Reference Study Group WHO Child Growth Standards: Length/Height-for-Age, Weight-for-Age, Weight-for-Length, Weight-for-Height and Body Mass Index-for-Age: Methods and Development. Geneva: World Health Organization (2006). Available online at: http://www.who.int/childgrowth/standards/Technical_report.pdf

[14] Brown KH. WHO/UNICEF review on complementary feeding and suggestions for future research: WHO/UNICEF guidelines on complementary feeding. Pediatrics. (2000) 106:1290. 11061836

[15] Montagnoli LC Barbieri MA Bettiol H Marques IL Souza Ld. Growth impairment of children with different types of lip and palate clefts in the first 2 years of life: a cross-sectional study. J Pediatr. (2005) 81:461–5. 10.2223/JPED.1420 16385363

[16] Lee J Nunn J Wright C. Height and weight achievement in cleft lip and palate. Arch Dis Child. (1997) 76:70–2. 10.1136/adc.76.1.70a 9059168 PMC1717033

